# Two Cases of Reflex Irritation Cured by Circumcision

**Published:** 1888-10

**Authors:** J. F. Cole

**Affiliations:** Carrollton, Ga.


### TWO CASES OF REFLEX IRRITATION CURED BY CIRCUMCISION.

BY J. F. COLE, M. D., CARROLLTON, GA.

CASE 1.—A boy, aged 14 years, was brought to my office for examination. Upon investigating I found he could not urinate freely; digestion bad; complained of gravel, which has existed from infancy; sallow color; cellular tissue seemed to be full of water; although his weight was only fifty pounds, locomotion bad. The urine would pass from the bladder and be retained in the long prepuce; the orifice was not larger than would allow a small pin's head to pass; the urine would only escape by drops. The little patient would have to press on the distended prepuce and force it away as one would using a syringe. I at once decided to operate and stated the facts as near as I could to his parents, to which they gave their assent. After he was anæsthetized I performed the little operation of circumcision.

Ten months after operation all urinary trouble was gone; weight increased to eighty pounds; in fact, he is well.

CASE 2.—On April 17th, 1884, Dr. A. B. Cole was called to Mrs. B's. to see a little boy, aged three years. On his arrival he found the child having convulsions about three hours apart; considerable excitement in circulation; temperature up; tongue coated. He at once made his diagnosis: worm fever and treated it for such.

On 18th, made another visit. Found his patient quiet; symptoms all better, except some rise in temperature, tongue remained furred.

In the decline of the first symptoms, it was discovered by its parents that it could not use its legs and remained so.

On May 24th, he was summoned again. Upon reaching the bedside of the patient, to his surprise he found that pareplegia had been existing ever since the first attack in April; also found it to have a troublesome cough, which could not be controlled by medicine.

At this visit he found the child would be attacked with acute pains in a foot, an eye, a finger, or its head; no part exempt from this symptom. It would not stay in one vicinity more than one or two minutes until it would go to some other part, and leave that member to rest only to wait its return.

The little patient's mind was not as good as one of that age. Its physician then examined its spine, and found it to be very tender, nearly the whole length. Then his diagnosis was, of course, spinal irritation, and treated it for such, with no permanent benefit.

On June 7th, he was told by the child's parents that it had gravel and could not urinate easy. The doctor then had his patient brought to my office in consultation with me. We concluded that the symptoms were reflex irritation and proceeded to examine for the cause. Upon approaching the penis, we found it with a long prepuce, the orifice large enough for the urine to escape only by drops. Of course, the remedy was to operate.

Twenty-four hours after the removal of the redundant prepuce, the child was playing in the yard without help or support in any way; paralysis gone; calculus symptoms passed away; spinal irritation removed; craziness left; in fact, all the above symptoms stricken out. The little fellow is now well.

I am of the opinion that we have many cases of paralysis and bladder troubles that we drug month after month and even years by overlooking that little member, the penis.

